# Dominant Myocardial Fibrosis and Complex Immune Microenvironment Jointly Shape the Pathogenesis of Arrhythmogenic Right Ventricular Cardiomyopathy

**DOI:** 10.3389/fcvm.2022.900810

**Published:** 2022-06-29

**Authors:** Wenzhao Lu, Yao Li, Yan Dai, Keping Chen

**Affiliations:** State Key Laboratory of Cardiovascular Disease, National Center for Cardiovascular Diseases, Arrhythmia Center, Fuwai Hospital, Chinese Academy of Medical Sciences & Peking Union Medical College, Beijing, China

**Keywords:** arrhythmogenic right ventricular cardiomyopathy, RNA sequencing, myocardial fibrosis, immune microenvironment, bioinformatics analysis

## Abstract

**Background:**

Arrhythmogenic right ventricular cardiomyopathy (ARVC) is a heritable life-threatening myocardial disease characterized by ventricular arrhythmias and sudden cardiac death. Few studies used RNA-sequencing (RNA-seq) technology to analyze gene expression profiles, hub genes, dominant pathogenic processes, immune microenvironment in ARVC. This study aimed to explore these questions *via* integrated bioinformatics analysis.

**Methods:**

RNA-sequencing datasets of GSE107475, GSE107311, GSE107156, and GSE107125 were obtained from the Gene Expression Omnibus database, including right and left ventricular myocardium from ARVC patients and normal controls. Weighted gene co-expression network analysis identified the ARVC hub modules and genes. Functional enrichment and protein-protein interaction analysis were performed by Metascape and STRING. Single-sample gene-set enrichment analysis (ssGSEA) was applied to assess immune cell infiltration. Transcription regulator (TF) analysis was performed by TRRUST.

**Results:**

Three ARVC hub modules with 25 hub genes were identified. Functional enrichment analysis of the hub genes indicated that myocardial fibrosis was the dominant pathogenic process. Higher myocardial fibrosis activity existed in ARVC than in normal controls. A complex immune microenvironment was discovered that type 2 T helper cell, type 1 T helper cell, regulatory T cell, plasmacytoid dendritic cell, neutrophil, mast cell, central memory CD4 T cell, macrophage, CD56dim natural killer cell, myeloid-derived suppressor cell, memory B cell, natural killer T cell, and activated CD8 T cell were highly infiltrated in ARVC myocardium. The immune-related hub module was enriched in immune processes and inflammatory disease pathways, with hub genes including CD74, HLA-DRA, ITGAM, CTSS, CYBB, and IRF8. A positive linear correlation existed between immune cell infiltration and fibrosis activity in ARVC. NFKB1 and RELA were the shared TFs of ARVC hub genes and immune-related hub module genes, indicating the critical role of NFκB signaling in both mechanisms. Finally, the potential lncRNA–miRNA–mRNA interaction network for ARVC hub genes was constructed.

**Conclusion:**

Myocardial fibrosis is the dominant pathogenic process in end-stage ARVC patients. A complex immune microenvironment exists in the diseased myocardium of ARVC, in which T cell subsets are the primary category. A tight relationship exists between myocardial fibrosis activity and immune cell infiltration. NFκB signaling pathway possibly contributes to both mechanisms.

## Introduction

As a life-threatening inherited myocardial disease, arrhythmogenic right ventricular cardiomyopathy (ARVC) is the common cause of malignant ventricular arrhythmias or sudden cardiac death (SCD) in young adults and athletes (1), characterized by progressive fibrofatty replacements of functional myocardium, predominantly the right ventricle (2, 3). The prevalence of ARVC is estimated to range from 1/5,000 to 1/2,000 (1).

Genetic research has found that ARVC is associated with gene mutations of cell junctions, especially the mutations in genes encoding desmosome components, including plakoglobin (JUP), desmoplakin (DSP), plakophilin2 (PKP2), desmoglein2 (DSG2), and desmocollin2 (DSC2) (4). Desmosome is the vital intercellular adhesion structure for maintaining the integrity and mechanical strength of myocardium (5). Currently, the pathogenic mechanisms related to desmosome dysfunction include cardiomyocyte detachment and loss (6, 7), interference to WNT/β-catenin (4, 8, 9) and Hippo/YAP signaling pathways (10–12) by nuclear-localized plakoglobins, and abnormal activation of TGF-β (7, 13, 14), PPARγ (8, 15, 16), and GSK3β (17, 18) signaling pathways, which have been found to cause fibrofatty replacements or myocardial fibrosis. Besides, inflammation or immune response is considered to participate in the disease process; it can occur secondary to cardiomyocyte death and damages cell junctions, releasing inflammatory cytokines that interfere with normal signaling pathways and promote myocardial fibrosis (19–21). Neutrophils, macrophages, T cells, mast cells, and B cells have been found to infiltrate in the fibrofatty-infiltrated area (21, 22).

Despite the accumulated knowledge of ARVC pathogenesis, ample space remains to explore. High-throughput RNA sequencing (RNA-seq) is a technology to obtain large-scale information about gene expression, making it possible to discover novel disease processes, biomarkers, and therapeutic targets (23). Whereas, few studies applied this technology to assess overall gene expression profiles, dominant pathological processes, and hub genes in ARVC. Besides, comprehensive analysis of the immune microenvironment is scarce despite the infiltration of several immune cell types has been found in ARVC myocardium (21). This study aimed to investigate these problems based on RNA-seq data and integrated bioinformatics approaches, such as weighted gene co-expression network analysis (WGCNA) and single-sample gene-set enrichment analysis (ssGSEA).

## Materials and Methods

### RNA-Sequencing Data

RNA-seq data was downloaded from the Gene Expression Omnibus (GEO)^1^ database. The research group released the data series on Nov. 20, 2020, including GSE107475 [9 samples of ARVC right ventricular myocardium (ARVC-RV)], GSE107311 [6 samples of ARVC left ventricular myocardium (ARVC-LV)], GSE107156 [5 samples of normal right ventricular myocardium (N-RV)], GSE107125 [6 samples of normal left ventricular myocardium (N-LV)]. These four series were the latest and largest RNA-seq dataset of explanted myocardial tissue from definitely diagnosed ARVC patients who underwent heart transplantation and non-diseased donor hearts in the GEO database, using the Illumina HiSeq 2500 (*Homo sapiens*) sequencing platform. Before weighted gene co-expression network analysis (WGCNA), the data was transformed by the robust quantile normalization and log2(n + 1).

### Weighted Gene Co-expression Network Analysis

weighted gene co-expression network analysis is an approach to finding co-expression modules based on gene expression levels, in which genes are highly correlated with each other. After data normalization and transformation, the top 75% genes of median absolute deviations (MADs) were enrolled for WGCNA. The network type was set to “signed hybrid” to highlight positive correlation while attenuating negative and zero correlations. The more robust biweight midcorrelation (bicor) was applied to calculate inter-gene correlations and construct the adjacency matrix. The soft power was picked up with the R-square threshold of 0.9 to construct a co-expression network satisfying the scale-free distribution. The minimum module size and the dendrogram cutting threshold were respectively set to 30 and 0.2 for module calculation and combination. According to the computer hardware, the maximum block size of 20000 was selected to calculate all the enrolled genes together instead of batch processes (24).

### Identification of Arrhythmogenic Right Ventricular Cardiomyopathy Hub Modules

Module-trait correlations were calculated between the module eigengenes (the first principal component of a given module) and the ARVC phenotype to identify the ARVC-related modules. According to tissue sources, seven trait assignments were developed: (1) ARVC-RV (ARVC-RV = 1, others = 0); (2) ARVC-LV (ARVC-LV = 1, others = 0); (3) N-RV (N-RV = 1, others = 0); (4) N-LV (N-LV = 1, others = 0); (5) ARVC (ARVC-RV/LV = 1, N-RV/LV = 0); (6) RV-LV (ARVC/N-RV = 1, ARVC/N-LV = 0); (7) Gradient (ARVC-RV = 4, ARVC-LV = 3, N-RV = 2, N-LV = 1) (Supplementary Table 1). Spearman’s correlation coefficients (SCC) with *P* values were calculated to assess the module-trait correlations (25). Modules positively correlated with the trait assignments of “ARVC-RV,” “ARVC-LV,” “ARVC,” “RV-LV,” and “Gradient” while negatively correlated with “N-RV” and “N-LV” were considered ARVC-related.

In order to identify the ARVC hub modules, the correlation score (Cor.score) and the significance score (Sig.score) were invented: Cor.score = [SCC_(ARVC–RV)_ + SCC_(ARVC–LV)_ + SCC_(ARVC)_ + SCC_(Gradient)_ + SCC_(RV–LV)_ – SCC_(N–RV)_ – SCC_(N–LV)_]/7; Sig.score = –log10{[P_(ARVC–RV)_ × P_(ARVC–LV)_ × P_(ARVC)_ × P_(Gradient)_ × PCC_(RV–LV)_ × P_(N–RV)_ × P_(N–LV)_]^(1/7)}. ARVC hub modules were defined as those with Cor.score ≥ 0.5 and Sig.score > 2.

### Identification of Arrhythmogenic Right Ventricular Cardiomyopathy Hub Genes

After identifying the ARVC hub modules, module membership (MM) and gene significance (GS) were calculated for each gene. Intra-modular candidate hub genes were defined as those with MM > 0.8 and GS > 0.5 (24, 25). Then the topological overlap matrix (TOM) of the candidate hub genes was obtained and converted to connectivity weights of each pair of genes. According to the connectivity weights, the top-1000 gene pairs were put into the Cytoscape software (version 3.7.2) to establish the weighted co-expression network while calculating the connection degree for each gene by the CytoHubba (version 0.1) plug-in. The top-50 genes of connection degrees (TOM-hub50) were selected. On the other hand, the protein-protein interaction (PPI) network of the candidate hub genes was obtained from the STRING database^2^ (26) and the top-50 proteins (genes) ranked by connection degrees (PPI-hub50) in the PPI network were fetched. The overlapped genes of TOM-hub50 and PPI-hub50 in each ARVC hub module were combined to explore their PPI network, in which the dominant gene cluster was recognized by the MCODE (version 1.6.1) plug-in as the ARVC hub genes (25).

### Immune Microenvironment Analysis and Immune-Related Hub Module

Single-sample gene-set enrichment analysis was applied to calculate the enrichment scores of 28 immune cell types for each sample, and a higher score represents a higher degree of immune cell infiltration (27, 28). Spearman’s correlation analysis was performed between the immune cell scores and the ARVC phenotype to identify the significantly and positively ARVC-related immune cells (SCC > 0, *P* < 0.05), which were considered highly infiltrated in ARVC myocardium. Subsequently, we analyzed the Pearson’s correlations between the modules and the highly infiltrated immune cells. The module most positively correlated with the total score of the highly infiltrated immune cells was considered as the immune-related hub module.

### Construct lncRNA–MiRNA–mRNA Network for Arrhythmogenic Right Ventricular Cardiomyopathy Hub Genes

Differentially expressed genes between ARVC and normal samples, defined as | log2(fold change)| > 1 and false discovery rate (FDR) < 0.05, were analyzed by the DEseq2 program. Differentially expressed lncRNAs within the ARVC hub modules were selected to calculate their Pearson’s correlations with the intra-modular candidate hub genes (mRNAs). Those with Pearson’s correlation coefficients (PCC) > 0.8 and *P* < 0.01 were considered the potentially interacted lncRNA-mRNA pairs. Then the interacted miRNAs of lncRNAs and mRNAs were searched in the RNAinter database^3^ (29) with confidence scores > 0.2. Hypergeometric test identified significant miRNA overlaps of lncRNA-mRNA pairs with FDR < 0.01. Pairs containing the ARVC hub genes were selected to construct the potential lncRNA–miRNA–mRNA network. The overlapped miRNAs of each lncRNA–mRNA pair were regarded as a miRNA cluster (miR-cluster), and the top-3 miRNAs of total confidence scores in each miR-cluster were selected to be presented. Topological network analysis was performed on the Cytoscape software (30, 31).

### Other Methods and Analytical Software

Functional enrichment analysis, including Gene ontology biological processes (GO-BP) and Kyoto Encyclopedia of Genes and Genomes (KEGG) pathways, was performed by Metascape^4^ (32). The enrichment score of a given gene set for each sample was calculated by ssGSEA; the higher the score is, the more up-regulated is the gene set (33). Transcription regulators (TFs) targeting given genes were searched in the TRRUST database^5^ (34), and those with the FDR < 0.05 were considered significantly enriched. Data analysis and visualization were completed by R (version 4.1.2) and RStudio (2021.09.2 + 382), accompanied by R packages including WGCNA (1.70-3), DESeq2 (1.34.0), GSVA (1.42.0), preprocessCore (1.56.0), pheatmap (1.0.12), and ggplot2 (3.3.5).

## Results

### Weighted Gene Co-expression Network Analysis Identified Arrhythmogenic Right Ventricular Cardiomyopathy Hub Modules

**FIGURE 1:**
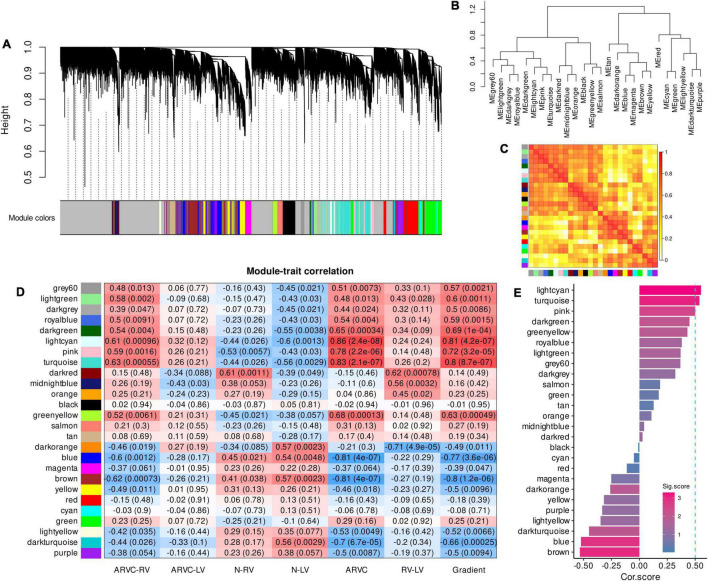
Co-expression gene modules produced by WGCNA and module-trait correlation analysis to select the ARVC hub modules. **(A)** The dendrogram illustrating modules generated by WGCNA; gray color refers to genes not clustered. **(B)** The hierarchical clustering tree of modules. **(C)** The heatmap of eigengene adjacency, darker red color indicated a closer relationship between two modules. **(D)** Correlations between module eigengenes and seven trait assignments. **(E)** Cor.score and Sig.score of all modules, modules with Cor.score ≥ 0.5 and Sig.score > 2 are regarded as ARVC hub modules. WGCNA, weighted gene co-expression network analysis; ARVC, arrhythmogenic right ventricular cardiomyopathy; Cor.score, correlation score; Sig.score, significance score.

A total of 26 samples with 19,005 genes were enrolled in WGCNA. A favorable scale-free co-expression network (R^2^ = 0.9) was constructed with the optimized soft power of 10 (Supplementary Figure 1), discovering 26 gene modules (Figure 1A, genes in each module were listed in Supplementary-Data-Sheets.Xlsx). The correlations between modules were demonstrated in Figures 1B,C.

The modules named grey60, lightgreen, darkgrey, royalblue, darkgreen, lightcyan, pink, and turquoise were positively correlated with the trait assignments of “ARVC-RV,” “ARVC-LV,” “ARVC,” and “Gradient” in general while being negatively correlated with “N-RV” and “N-LV” (Figure 1D). Moreover, the modular-trait correlation patterns of “ARVC-RV” and “ARVC-LV” were similar, but the levels of “ARVC-LV” were milder than “ARVC-RV” while they were both significantly distinct from normal controls (Figure 1D).

After calculating Cor.score and Sig.score of each module, the lightcyan (Cor.score = 0.55, Sig.score = 3.29), turquoise (Cor.score = 0.54, Sig.score = 3.07), and pink (Cor.score = 0.50, Sig.score = 2.53) modules satisfied the definition of ARVC hub modules (Figure 1E) and were highly correlated with each other (Figure 1C), containing 247, 524, and 1667 genes, respectively (Supplementary-Data-Sheets.Xlsx).

### Identification and Functional Analysis of Arrhythmogenic Right Ventricular Cardiomyopathy Hub Genes

**FIGURE 2:**
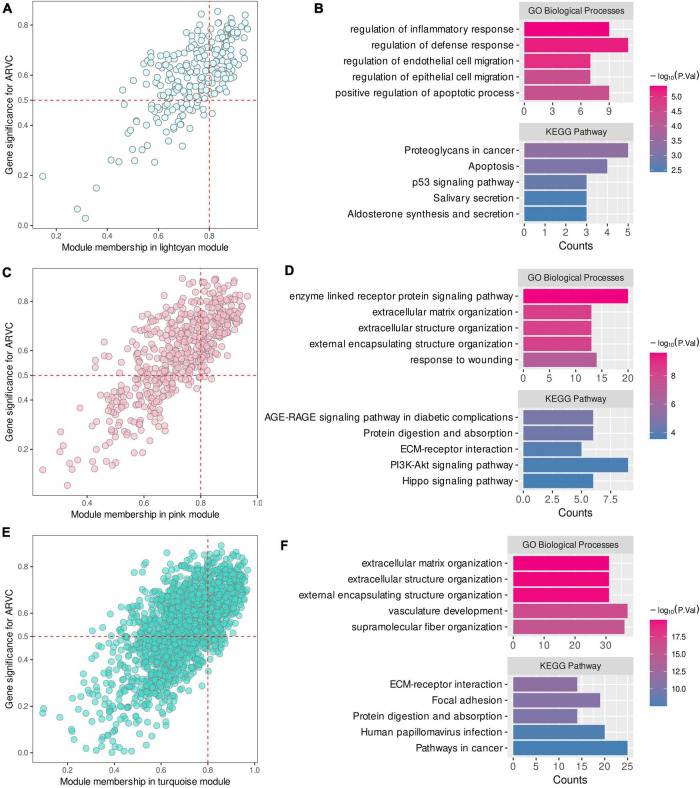
Identification of intra-modular candidate hub genes in ARVC hub modules. **(A,B)** Candidate hub genes in the lightcyan module with functional enrichment results. **(C,D)** Candidate hub genes in the pink module with functional enrichment results. **(E,F)** Candidate hub genes in the turquoise module with functional enrichment results.

Module membership and GS of genes in the lightcyan, pink, and turquoise modules were calculated. According to the definition of candidate hub genes (MM > 0.8 and GS > 0.5), there were 95, 172, and 390 candidate hub genes in the three modules, respectively (Figures 2A,C,E). Functional enrichment analysis for the candidate hub genes revealed the critical functions of the three modules (detailed results are provided in Supplementary-Data-Sheets.Xlsx). The lightcyan module was mainly associated with inflammatory and defense response regulation, endothelial and epithelial cell migration, positive apoptosis regulation, proteoglycans in cancer, and P53 signaling pathway (Figure 2B). The pink module was associated with extracellular matrix (ECM) and structure organization, enzyme-linked receptor protein signaling pathway, response to wounding, cellular adhesion and migration (ECM–receptor interaction) (Figure 2D). For the turquoise module, the primary functions were similar to the pink module, including ECM organization, vasculature development, fiber organization, cellular adhesion and migration (ECM–receptor interaction and focal adhesion pathway) (Figure 2F).

**FIGURE 3:**
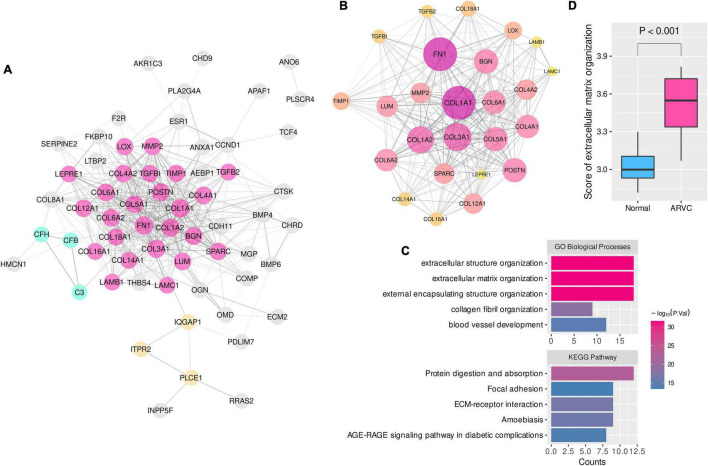
The PPI network and enrichment analysis of ARVC hub genes. **(A)** The PPI network of 62 candidate hub genes from the three ARVC hub modules (disconnected nodes were hided). **(B)** The primary cluster with 25 ARVC hub genes in the PPI network, larger node sizes represent higher connection degrees within the network. **(C)** Functional enrichment of the 25-hub-gene cluster. **(D)** Comparison of the ssGSEA score of extracellular matrix organization between ARVC and normal samples. ssGSEA, single sample gene-set enrichment analysis. GO-BP, Gene Ontology biological process.

Consequently, 25, 19, and 18 candidate hub genes were identified by overlapping the TOM-hub50 and the PPI-hub50 in the lightcyan, pink, and turquoise modules, respectively (Table 1). Due to the close linkage between the hub modules, these 62 candidate hub genes were combined to investigate their PPI network (Figure 3A), in which the MCODE plug-in identified a 25-hub-gene cluster (Figure 3B). The top-5 genes ranked by connection degrees in this cluster were COL1A1, FN1, COL3A1, COL1A2, COL5A1. Enrichment analysis demonstrated that these 25 hub genes were associated with ECM or extracellular structure organization, collagen fibril organization, blood vessel development, cellular adhesion and migration (ECM–receptor interaction and focal adhesion pathway) (Figure 3C).

**TABLE 1:** TOM-hub50 & PPI-hub50 overlapped genes in three ARVC hub modules.

ARVC-related modules	TOM-hub50 and PPI-hub50 overlapped genes
Lightcyan (n = 25)	ESR1, LUM, LOX, OGN, APAF1, ITPR2, MGP, TGFBI, OMD, PLA2G4A, AKR1C3, ECM2, ANO6, ANXA1, CFB, HMCN1, CFH, CHD9, CTSK, PLSCR4, RAB23, PCSK5, SGCE, TCF4, CCDC102B
Pink (n = 19)	FN1, POSTN, COL4A1, COL12A1, BGN, COL18A1, TGFB2, LTBP2, SERPINE2, THBS4, PDLIM7, F2R, INPP5F, AEBP1, PLCE1, COMP, BMP6, RASL11B, RRAS2
Turquoise (n = 18)	COL1A1, COL3A1, COL1A2, COL6A1, COL5A1, COL6A2, MMP2, SPARC, COL4A2, BMP4, COL14A1, LAMB1, LEPRE1, COL16A1, CDH11, IQGAP1, FKBP10, COL8A1

*TOM-hub50, top-50 candidate hub genes ranked by connection degrees in the co-expression network established by the top-1,000 gene pairs of weights in the topological overlap matrix produced by WGCNA. PPI-hub50, top-50 candidate hub genes based on the protein-protein interaction network.*

These results indicated that myocardial fibrosis, represented by the ECM organization process, might play a dominant role in the pathogenesis of ARVC. The ssGSEA enrichment scores of the GO-BP item “extracellular matrix organization” were calculated to quantify the fibrosis activity for each sample. Inter-group comparison further confirmed the higher fibrosis activity in the ARVC samples than the normal controls (Figure 3D).

### Immune Microenvironment and Immune-Related Hub Module in Arrhythmogenic Right Ventricular Cardiomyopathy

**FIGURE 4:**
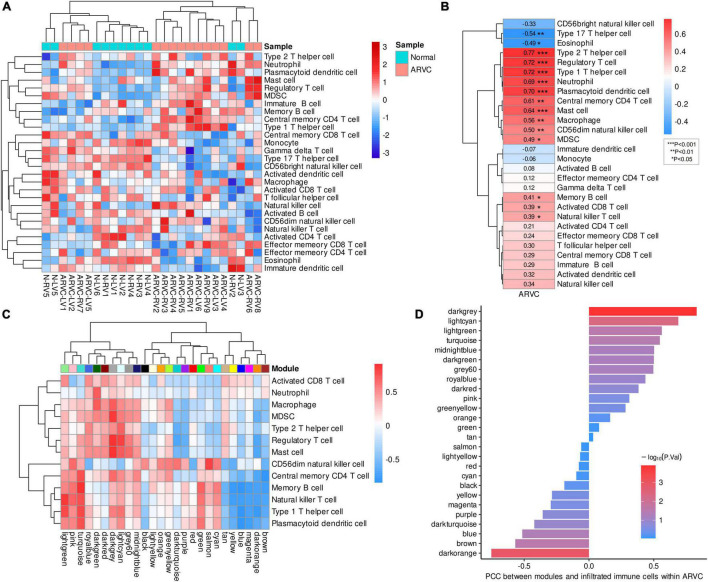
Immune cell infiltration and immune-module correlation analysis. **(A)** Heatmap of the ssGSEA scores of 28 types of immune cells with inter-sample standardization, a higher score means the immune cell is highly infiltrated. **(B)** Correlation between ARVC phenotype and immune cell scores. **(C)** Correlation analysis between modules and the highly infiltrated immune cells within ARVC samples. **(D)** Correlation between moduless and the total score of the highly infiltrated immune cells within ARVC samples. MDSC, myeloid-derived suppressor cells; PCC, Pearson’s correlation coefficient.

After calculating the enrichment scores of 28 immune cell types for each sample, clustering analysis revealed a discrepancy in infiltration patterns between ARVC and normal samples (Figure 4A). Spearman’s correlation analysis indicated that ARVC phenotype was significantly and positively correlated with the scores of type 2 T helper cell (Th2), type 1 T helper cell (Th1), regulatory T cell (Treg), plasmacytoid dendritic cell (PDC), neutrophil, mast cell, central memory CD4 T (T_CM_) cell, macrophage, CD56dim natural killer (NK) cell, myeloid-derived suppressor cell (MDSC), memory B cell (MBC), natural killer T (NKT) cell, and activated CD8 T cell (Figure 4B). These 13 positively ARVC-related immune cells were considered highly infiltrated.

**FIGURE 5:**
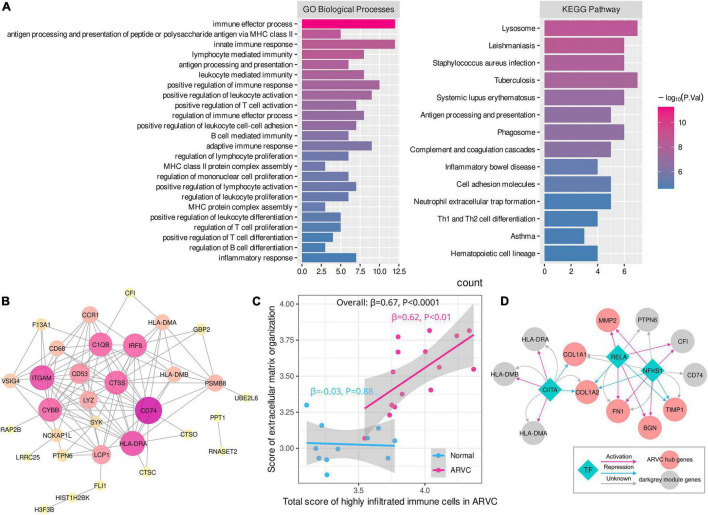
Functional enrichment and PPI network of the immune-related module and the relationship between myocardial fibrosis and immune cell infiltration. **(A)** Functional enrichment of genes in darkgrey module. **(B)** PPI network of genes in darkgrey module, larger node sizes represent higher connection degrees within the network (disconnected nodes were concealed). **(C)** The linear correlation analysis between the score of extracellular matrix organization and the total score of the highly infiltrated immune cells. **(D)** Shared transcription regulators (TFs) of ARVC hub genes and darkgrey-module genes. MHC, class II major histocompatibility complex; Th1, type 1 T helper cell; Th2, type 2 T helper cell.

Pearson’s correlations between gene modules and these highly infiltrated immune cells were analyzed and grouped by hierarchical clustering within the 15 ARVC samples, indicating that the modules of lightgreen, pink, turquoise, royalblue, darkgreen, darkred, darkgrey, lightcyan, grey60, and midnightblue were more positively correlated with the immune cells in general, among which there were two clusters: one cluster (lightgreen, pink, and turquoise modules) possessed the stronger correlations with CD56dim NK cell, T_CM_ cell, MBC, NKT cell, Th1, and PDC; another one (royalblue, darkgreen, darkred, darkgrey, lightcyan, grey60, and midnightblue modules) was correlated with activated CD8 T cell, neutrophil, macrophage, MDSC, Th2, Treg, and mast cell more substantially (Figure 4C). Since the darkgrey module possessed the highest positive correlation with the total score of the highly infiltrated immune cells (Figure 4D), it was regarded as the immune-related hub module, which was proved by functional analysis that the intra-modular genes participate in various processes of immunoregulation and signaling pathways of infectious, inflammatory or autoimmune diseases (Figure 5A, Supplementary-Data-Sheets.Xlsx). The PPI network of the darkgrey module was shown in Figure 5B, among which the top-10 genes ranked by connection degrees were CD74, HLA-DRA, ITGAM, CTSS, CYBB, IRF8, C1QB, CD53, LCP1, and LYZ (Figure 5B).

Furthermore, linear regression analysis demonstrated that the total score of the highly infiltrated immune cells in ARVC was significantly and positively correlated with the enrichment score of “extracellular matrix organization” (β = 0.62, *P* < 0.01), while the fibrosis activity in normal myocardium was at a low level and had no significant correlation with the extent of immune cell infiltration (β = 0.03, *P* = 0.88) (Figure 5C). TF analysis by the TRRUST database identified 16 and 14 key TFs for the ARVC hub genes and the darkgrey module genes, respectively (Tables 2, 3), among which NFKB1, CIITA, and RELA were three shared TFs (Figure 5D).

**TABLE 2:** Key transcription regulators (TF) targeting the 25 ARVC hub genes.

Key TF	Target genes	P value	FDR
RELA	COL1A2, MMP2, TIMP1, COL1A1, BGN, FN1 (n = 6)	1.65E-06	1.37E-05
NFKB1	BGN, TIMP1, COL1A2, MMP2, FN1, COL1A1 (n = 6)	1.72E-06	1.37E-05
TWIST2	POSTN, FN1, MMP2 (n = 3)	4.08E-06	2.18E-05
ATF2	FN1, TGFB2, MMP2 (n = 3)	8.75E-06	3.50E-05
TWIST1	TIMP1, MMP2, FN1 (n = 3)	1.15E-05	3.69E-05
VHL	COL4A2, SPARC (n = 2)	0.0003	0.0008
SP3	TIMP1, COL1A1, MMP2 (n = 3)	0.0004	0.0009
NFIC	COL18A1, COL1A1 (n = 2)	0.0005	0.0011
CIITA	COL1A1, COL1A2 (n = 2)	0.0007	0.0013
STAT6	COL1A2, COL1A1 (n = 2)	0.00095	0.0015
MYB	COL1A1, COL1A2 (n = 2)	0.001	0.0015
SP1	MMP2, TIMP1, COL18A1, COL1A1 (n = 4)	0.0028	0.0037
TFAP2A	MMP2, COL1A1 (n = 2)	0.0037	0.0045
HIF1A	MMP2, LOX (n = 2)	0.0050	0.0057
YY1	COL1A2, POSTN (n = 2)	0.0059	0.0063
STAT3	TIMP1, MMP2 (n = 2)	0.0139	0.0139

*FDR, false discovery rate.*

**TABLE 3:** Key transcription regulators (TF) targeting the darkgrey module genes.

Key TF	Target genes	P value	FDR
SPI1	CYBB, SCARB2, ITGAM, FLI1, CD68, CTSS (n = 5)	1.08E-08	1.51E-07
ELF1	CD68, CYBB, FLI1 (n = 3)	1.28E-06	8.98E-06
RFXANK	HLA-DMA, HLA-DRA, HLA-DMB (n = 3)	6.88E-06	2.41E-05
RFXAP	HLA-DMA, HLA-DRA, HLA-DMB (n = 3)	6.88E-06	2.41E-05
RFX5	HLA-DRA, HLA-DMB, HLA-DMA (n = 3)	1.23E-05	3.44E-05
CIITA	HLA-DMA, HLA-DRA, HLA-DMB (n = 3)	6.60E-05	0.000154
IRF8	CYBB, CD68 (n = 2)	0.0003	0.0007
GATA1	FLI1, F13A1, CYBB (n = 3)	0.0004	0.0007
RFX1	HLA-DMB, HLA-DRA (n = 2)	0.0007	0.001
HDAC1	HLA-DRA, CLDN7 (n = 2)	0.0141	0.0197
ETS1	F13A1, FLI1 (n = 2)	0.0172	0.0219
STAT1	CCR1, IRF8 (n = 2)	0.0193	0.0226
RELA	CD74, CFI, PTPN6 (n = 3)	0.041	0.0417
NFKB1	PTPN6, CD74, CFI (n = 3)	0.0417	0.0417

*FDR, false discovery rate.*

### LncRNA–MiRNA–mRNA Network for Arrhythmogenic Right Ventricular Cardiomyopathy Hub Genes

**FIGURE 6:**
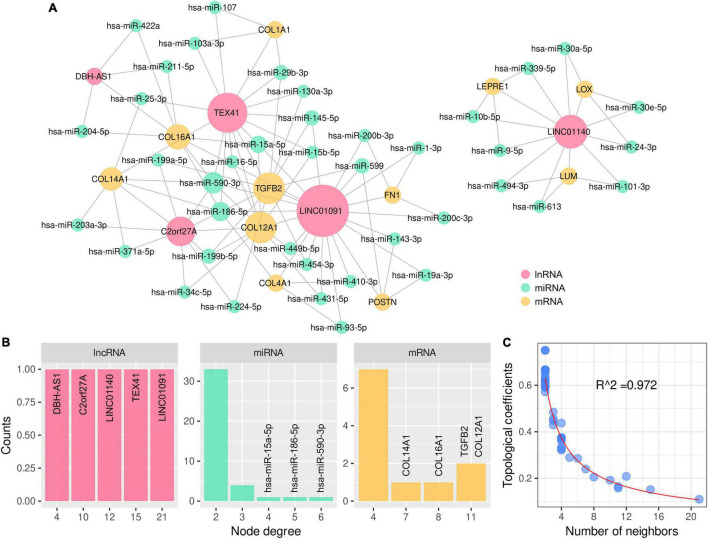
LncRNA–miRNA–mRNA network for 11 ARVC hub genes with topological analysis. **(A)** The complete lncRNA-miRNA-mRNA network. **(B)** The distributions of the node degrees of lncRNA, miRNA, and mRNA, respectively. **(C)** The distribution of the topological coefficients indicating the favorable fitness of scale-free network features.

A total of 198 differentially expressed lncRNAs between ARVC and normal samples were identified, including 3 lncRNA in the lightcyan module, 27 in the pink module, and 52 in the turquoise module. Correlation analysis between these 82 lncRNAs and the candidate hub genes within the three modules discovered 934 lncRNA–mRNA pairs with PCC > 0.8 and *P* < 0.01, among which 258 pairs had significant miRNA overlaps (FDR < 0.01). Eventually, we discovered 17 significantly correlated lncRNA–mRNA pairs containing 11 ARVC hub genes and 17 corresponding miR-clusters (Table 4 and Supplementary-Data-Sheets.Xlsx). Sixteen miR-clusters had shared miRNAs (Supplementary Figure 2B). The lncRNA-miRcluster-mRNA network was constructed according to lncRNA-mRNA relationships and miR-cluster overlaps (Supplementary Figure 2A), satisfying the scale-free network distribution (R^2^ = 0.917) (Supplementary Figure 2D). Then the lncRNA-miRNA-mRNA network was constructed (Figure 6A), perfectly satisfying the scale-free distribution (R^2^ = 0.972) (Figure 6C). Ranked by the connection degree, the top-3 lncRNAs were LINC01091, TEX41, and LNIC01140; the top-4 mRNAs were TGFB2, COL12A1, COL16A1, and COL14A1; the top-3 miRNAs were hsa-miR-590-3p, hsa-miR-186-5p, and hsa-miR-15a-5p (Table 4 and Figure 6B).

**TABLE 4:** Significantly correlated and overlapped lncRNA-mRNA pairs with overlapped miRNAs.

LncRNA	mRNA	lncRNA–miRNA counts	mRNA–miRNA counts	Overlap miR-cluster	FDR	Top 3 miRNA
C2orf27A	COL12A1§	8	152	miR-cluster1 (n = 4)	0.003	hsa-miR-34c-5p hsa-miR-590-3p^‡^ hsa-miR-199b-5p
LINC01091*	COL12A1§	58	152	miR-cluster2 (n = 28)^†^	3.7E-16	hsa-miR-431-5p hsa-miR-449b-5p hsa-miR-15a-5p^‡^
TEX41*	COL12A1§	51	152	miR-cluster3 (n = 27)	7.3E-17	hsa-miR-15a-5p^‡^ hsa-miR-15b-5p hsa-miR-16-5p
C2orf27A	COL14A1§	8	69	miR-cluster4 (n = 3)	0.003	hsa-miR-203a-3p hsa-miR-371a-5p hsa-miR-590-3p^‡^
TEX41*	COL14A1§	51	69	miR-cluster5 (n = 10)	1.5E-05	hsa-miR-25-3p hsa-miR-590-3p^‡^ hsa-miR-186-5p^‡^
DBH-AS1	COL16A1§	15	39	miR-cluster6 (n = 3)	0.004	hsa-miR-422a hsa-miR-204-5p hsa-miR-211-5p
LINC01091*	COL16A1§	58	39	miR-cluster7 (n = 7)^†^	0.0002	hsa-miR-590-3p^‡^ hsa-miR-15a-5p^‡^ hsa-miR-186-5p^‡^
TEX41*	COL1A1	51	110	miR-cluster8 (n = 9)	0.003	hsa-miR-29b-3p hsa-miR-107 hsa-miR-103a-3p
LINC01091*	COL4A1	58	194	miR-cluster9 (n = 26)^†^	1.2E-11	hsa-miR-93-5p hsa-miR-186-5p^‡^ hsa-miR-410-3p
LINC01091*	FN1	58	137	miR-cluster10 (n = 16)	2.5E-06	hsa-miR-1-3p hsa-miR-200c-3p hsa-miR-200b-3p
LINC01140	LEPRE1	88	60	miR-cluster11 (n = 14)	4.5E-07	hsa-miR-339-5p hsa-miR-9-5p hsa-miR-10b-5p
LINC01140	LOX	88	86	miR-cluster12 (n = 30)^†^	8.9E-20	hsa-miR-30a-5p hsa-miR-30e-5p hsa-miR-24-3p
LINC01140	LUM	88	67	miR-cluster13 (n = 26)	2.3E-18	hsa-miR-101-3p hsa-miR-494-3p hsa-miR-613
LINC01091*	POSTN	58	66	miR-cluster14 (n = 14)	6.6E-09	hsa-miR-19a-3p hsa-miR-599 hsa-miR-143-3p
C2orf27A	TGFB2§	8	176	miR-cluster15 (n = 5)	0.0004	hsa-miR-199b-5p hsa-miR-224-5p hsa-miR-199a-5p
LINC01091*	TGFB2§	58	176	miR-cluster16 (n = 32)^†^	1.3E-18	hsa-miR-599 hsa-miR-454-3p hsa-miR-145-5p
TEX41*	TGFB2§	51	176	miR-cluster17 (n = 27)	3.1E-15	hsa-miR-29b-3p hsa-miR-130a-3p hsa-miR-145-5p

*Top-2 lncRNA ranked by node degrees.

*^§^Top-4 mRNA ranked by node degrees.*

*^†^Top-5 miR-clusters ranked by node degree.*

*^‡^Top-3 miRNA ranked by node degrees.*

*miR-cluster, cluster of overlapped miRNAs; FDR, false discovery rate.*

## Discussion

As the first bioinformatics study based on the latest and largest myocardial RNA-seq data from ARVC patients and non-diseased controls in the GEO database, there are several primary findings. (1) Myocardial fibrosis is the dominant pathogenic process in end-stage ARVC patients undergoing heart transplantation. (2) Th2, Th1, Treg, PDC, neutrophil, mast cell, TCM cell, macrophage, CD56dim NK cell, MDSC, MBC, NKT cell, and activated CD8 T cell are highly infiltrated in ARVC myocardium, constituting the complex immune microenvironment. (3) The immune-related hub module was identified and confirmed to be related to various immune functions or signaling pathways. (4) A positive linear correlation between immune cell infiltration and myocardial fibrosis in ARVC myocardium was detected. NFKB1, CIITA, and RELA were identified as the shared TFs of the ARVC hub genes and the immune-related hub module genes, among which NFKB1 and RELA encode components of the nuclear factor kappa B (NFκB) complex, suggesting the critical role of the NFκB signaling in both mechanisms.

WGCNA was applied to summarize the overall gene expression profiles in the format of co-expression modules, identifying the hub modules most positively correlated with the ARVC phenotype. Module-trait correlation analysis comprehensively reflected the correlation patterns between modules and different tissue sources, conquering the shortcomings of the conventional differential expression analysis (35). Eventually, three ARVC hub modules were recognized with their corresponding candidate hub genes. Functional enrichment analysis revealed that the pink and turquoise modules are dominantly correlated with ECM or collagen fiber organization. Notably, the pink module is associated with the Hippo signaling pathway, which has been reported to participate in the pathogenesis of ACM, as its activation suppresses the expression of genes associated with myocardial survival and growth while promoting the transcription of pro-apoptotic and adipogenic genes (12, 36, 37). Besides, activated Hippo signaling can repress the WNT/β-catenin pathway, prompting myocardial apoptosis and fibrofatty replacements (10, 11). “Pathways in cancer” was significantly enriched in the turquoise module, containing signaling pathways such as WNT, TGF-β, PPARγ, and cytokines, whose roles have been studied in ACM (4). Unlike the other two modules, functional diversity was discovered in the lightcyan module, such as inflammatory response, cell migration, pro-apoptosis, ECM organization, and P53 signaling pathway, which also work in ACM (2, 4, 21, 38).

Based on the topological features in the weighted co-expression network and the PPI network of the candidate hub genes, 25 hub genes were eventually identified, coding ECM components and participating in fibrogenic processes. *Via* calculating the ssGSEA score of ECM organization, we confirmed a significantly higher fibrosis activity in ARVC myocardium than normal controls. Among the hub genes, COL1A1, COL1A2, COL3A1, COL6A1, COL6A2, COL5A1, COL4A1, COL4A2, COL12A1, COL16A1, COL18A1, and COL14A1 encode the components of collagen fibril, the central part of ECM (39). FN1 encodes fibronectin 1, an element of ECM related to cell adhesion and migration (40). POSTN encodes periostin, a secreted ECM protein, playing a role in wound healing and post-infarction ventricular remodeling (41). BGN and SPARC participate in ECM organization in various tissues (42, 43), and BGN (biglycan) also plays a role in inflammation (44, 45). MMP2 and TIMP1 respectively encode matrix metalloproteinase 2 and tissue inhibitor of metalloproteinases 1, necessary for ECM cleaving, especially the collagen fibril (46, 47). Productions of LOX, LUM, and LEPRE1 also play a role in collagen synthesis and assembly (44, 48, 49). LAMB1 and LAMC1 encode subunits of laminin, the central non-collagenous element of basement membranes, participating in cell adhesion, migration, and differentiation (50). TGFB1 and TGFB2 encode transforming growth factor β superfamily members; previous studies have discovered the upregulation of TGF-β and activation of its downstream SMADs and MAPKs in ACM myocardium (13), JUP-deficient animals (7), and PKP2-deficient cell models (14), promoting ECM synthesis and repressing MMPs (51). Unlike the previous microarray study finding no up-regulated fibrogenic genes in ARVC myocardium (35), our findings revealed that myocardial fibrosis is the dominant pathogenic change in ARVC, and the collagen fibril is the main component accompanied by various enzymes and signaling molecules participating in fibrosis regulation. This discrepancy is likely due to the advancement in transcriptomics and bioinformatics technologies and the different collection and preparation of myocardial tissue. Still, it is reasonable to consider the results from the latest RNA-seq data more convincing.

Immune responses have been proposed to shape the pathogenesis of ACM. Previous studies have found immune cell infiltration in the regions with fibrofatty replacements by conventional histopathology, immunohistochemistry, and electron microscopy (20, 21). These studies discovered several types of immune cells instead of more detailed subsets. We used ssGSEA to calculate the enrichment scores of 28 immune cell types for each sample, reflecting their infiltration levels. Clustering and correlation analysis discovered 13 immune cell types highly infiltrated in ARVC myocardium, among which T cell subsets occupied the maximal proportion, indicating the essential role of T cell-mediated immunity in the immunologic mechanism of ARVC. Th1 produces interferon-γ (IFN-γ) that stimulates CD8+ T cells, macrophages, and B cells, mainly modulating cellular immunity (52). Th2 secretes IL-4, IL-5, and IL-13, mediating classical type 2 immune response (52). Treg prevents excessive immune responses and maintains the homeostasis of the immune system (52). NKT cells and activated CD8+ T cells are the effector cells of cellular immunity, executing cytotoxic reactions (53). T_CM_ cells mediate secondary cellular immunity, differentiating into effector T cells when encountering the previously remembered antigens (53). Other immune cell types include PDC, neutrophil, mast cell, macrophage, CD56dim NK cell, MBC, and MDSC, functioning in antigen presentation, phagocytosis, cytotoxic reaction, secondary immune response, and immunoregulation (54–56). Previous studies divided the inflammatory response into several phases in ACM murine models (20). In the early phase, neutrophil is the primary immune cell type, followed by accumulating macrophages and T cells that persist in the chronic phase. NKT cell, CD56dim NK cell, PDC, MDSC, and particular T cell subsets have not been studied and how they shape the pathogenesis in ARVC remains to explore. The diversity of the infiltrated immune cells implicates a more complicated immunologic mechanism than what has been elucidated.

Further, the darkgrey module was identified as the immune-related hub module, which was confirmed by functional enrichment analysis. The module was significantly linked to various immune processes and pathways, including T cell-mediated immunity, B cell-mediated immunity, innate immunity, antigen processing and presentation, immune cell adhesion, migration, activation, and proliferation regulation. Ten intra-modular hub genes were selected through PPI network analysis, which have never been reported in ARVC. CD74 and HLA-DRA, expressed in antigen-presenting cells such as B cell, dendritic cell, and macrophage, participate in antigen presentation to CD4+ T cells mediated by the class II major histocompatibility complex (MHC) (57, 58). CTSS produces a lysosomal cysteine proteinase that degrades protein antigens to peptides presented by MHC II molecules and remodels ECM components, working in both immune response and fibrosis (59). ITGAM encodes a subunit of integrin that facilitates the adhesion of neutrophils and monocytes to stimulated endothelium (60). CYBB encodes the β-chain of cytochrome B, an essential component of the microbicidal oxidase system in phagocytes (61). IRF8 is a transcription factor required for antigen capture, processing and presentation, and responses to cytokines including IFN-γ and IFN-β (62).

As the two critical pathogenic processes in ARVC, the relationship between myocardial fibrosis and immune cell infiltration is unclear. ECM interacts with immune cells, regulating their adhesion, migration, differentiation, and proliferation (63, 64). This study discovered a positive linear correlation between fibrosis activity and immune infiltration in ARVC myocardium, suggesting a close link between these two mechanisms. Subsequently, we found three shared TFs between the ARVC hub genes and the immune-related hub module genes. NFKB1 and RELA encode the components of the NFκB complex, a transcription regulator activated by signals like IL-1, tumor necrosis factors, Toll-like receptor, and CD40 ligand, playing an essential role in immune responses (65). NFκB signaling pathway has been found activated in ACM and is closely linked to GSK3β signaling, which promoted TGF-β1 expression and enhanced its downstream signaling, leading to myocardial fibrosis (21). Consequently, it is reasonable to believe that NFκB signaling pathway simultaneously functions in myocardial fibrosis and immune response in ARVC, which may be the potential intervention target. A study found that repressing NFκB signaling could prevent the development of ACM features, such as redistribution of plakoglobin, Cx43, and GSK3β, myocardium apoptosis, and releases of inflammatory cytokines (18).

Finally, we constructed the potential lncRNA–miRNA–mRNA regulatory network for the ARVC hub genes, which was discovered as an epigenetic regulatory mechanism in cardiovascular diseases (66). Three key lncRNAs were identified, among which LINC01091 and TEX41 interact with mRNAs of ECM components and TGFB2, while LINC01140 interacts with mRNAs of ECM-modulating proteins.

### Limitations

Due to the lack of additional clinical phenotype information such as sex, age, survival time, ventricular arrhythmic events, and sudden cardiac death, it is challenging to analyze hub genes or biomarkers correlated with these critical clinical conditions. The results were based exclusively on bioinformatics analysis, requiring further experimental confirmations. Still, they provide some new insights into ARVC pathogenesis and references for future research. Finally, since the insufficient miRNA sequencing data, the lncRNA–miRNA–mRNA network was established based on the correlations and the interacted miRNA overlaps between lncRNAs and mRNAs. Hence the lncRNA–miRNA–mRNA network is theoretical and requires experimental validations.

## Conclusion

Myocardial fibrosis is the dominant pathogenic process in end-stage ARVC patients. A complex immune microenvironment exists in the diseased myocardium of ARVC, in which T cell subsets are the primary immune cell type. A close relationship exists between myocardial fibrosis activity and immune cell infiltration. NFκB signaling pathway possibly contributes to both mechanisms and is considered the potential intervention target. The feasibility of anti-fibrosis and immune-modulating therapies for ARVC remains to explore in the future.

## Data Availability Statement

Publicly available datasets were analyzed in this study. This accession numbers are here: GSE107475, GSE107311, GSE107156, and GSE107125.

## Author Contributions

WL and YL completed the data collection and preparation and drafted the manuscript. WL performed the data analysis and result interpretation. KC and YD performed the final approval of the submitted version. All authors participated in article revisions.

## Conflict of Interest

The authors declare that the research was conducted in the absence of any commercial or financial relationships that could be construed as a potential conflict of interest.

## Publisher’s Note

All claims expressed in this article are solely those of the authors and do not necessarily represent those of their affiliated organizations, or those of the publisher, the editors and the reviewers. Any product that may be evaluated in this article, or claim that may be made by its manufacturer, is not guaranteed or endorsed by the publisher.

## Footnotes

1
https://www.ncbi.nlm.nih.gov/geo/

2
https://string-db.org/

3
http://www.rnainter.org/

4
https://metascape.org/

5
https://www.grnpedia.org/trrust/
