# Supplementary material for: Dominant Myocardial Fibrosis and Complex Immune Microenvironment Jointly Shape the Pathogenesis of Arrhythmogenic Right Ventricular Cardiomyopathy
Source: Front Cardiovasc Med. 2022 Jun 29;9:900810. doi: 10.3389/fcvm.2022.900810 (PMC9278650; doi:10.3389/fcvm.2022.900810)
Supplement: Supplementary file 2 [file Data_Sheet_2.PDF]

Supplementary Table S1. Assignment conditions of samples

Samples	Assignment conditions						
	ARVC-RV	ARVC-LV	N-RV	N-LV	ARVC	RV-LV	Gradient
ARVC-RV1	1	0	0	0	1	1	4
ARVC-RV2	1	0	0	0	1	1	4
ARVC-RV3	1	0	0	0	1	1	4
ARVC-RV4	1	0	0	0	1	1	4
ARVC-RV5	1	0	0	0	1	1	4
ARVC-RV6	1	0	0	0	1	1	4
ARVC-RV7	1	0	0	0	1	1	4
ARVC-RV8	1	0	0	0	1	1	4
ARVC-RV9	1	0	0	0	1	1	4
ARVC-LV1	0	1	0	0	1	0	3
ARVC-LV2	0	1	0	0	1	0	3
ARVC-LV3	0	1	0	0	1	0	3
ARVC-LV4	0	1	0	0	1	0	3
ARVC-LV5	0	1	0	0	1	0	3
ARVC-LV6	0	1	0	0	1	0	3
N-RV1	0	0	1	0	0	1	2
N-RV2	0	0	1	0	0	1	2
N-RV3	0	0	1	0	0	1	2
N-RV4	0	0	1	0	0	1	2
N-RV5	0	0	1	0	0	1	2
N-LV1	0	0	0	1	0	0	1
N-LV2	0	0	0	1	0	0	1
N-LV3	0	0	0	1	0	0	1
N-LV4	0	0	0	1	0	0	1
N-LV5	0	0	0	1	0	0	1
N-LV6	0	0	0	1	0	0	1

ARVC, arrhythmogenic right ventricular cardiomyopathy; ARVC-RV, ARVC right ventricular myocardium; ARVC-LV, ARVC left ventricular myocardium; N-RV, normal right ventricular myocardium; N-LV, normal left ventricular myocardium.

Supplementary Table S2. Connection degree distribution of the lncRNA-miRNA-mRNA network

Symbols	Counts	Node degree
lncRNA		
LINC01091	1	21
TEX41	1	15
LINC01140	1	12
C2orf27A	1	10
DBH-AS1	1	4
mRNA		
TGFB2, COL12A1	2	11
COL16A1	1	8
COL14A1	1	7
POSTN, LUM, LOX, LEPRE1, FN1, COL4A1, COL1A1	7	4
miRNA		
hsa-miR-590-3p	1	6
hsa-miR-186-5p	1	5
hsa-miR-15a-5p	1	4
hsa-miR-145-5p, hsa-miR-599, hsa-miR-29b-3p, hsa-miR-199b-5p	4	3
hsa-miR-25-3p, hsa-miR-130a-3p, hsa-miR-454-3p, hsa-miR-199a-5p hsa-miR-224-5p, hsa-miR-143-3p, hsa-miR-19a-3p, hsa-miR-613 hsa-miR-494-3p, hsa-miR-101-3p, hsa-miR-24-3p, hsa-miR-30e-5p hsa-miR-30a-5p, hsa-miR-10b-5p, hsa-miR-9-5p, hsa-miR-339-5p hsa-miR-200b-3p, hsa-miR-200c-3p, hsa-miR-1-3p, hsa-miR-410-3p hsa-miR-93-5p, hsa-miR-103a-3p, hsa-miR-107, hsa-miR-211-5p hsa-miR-204-5p, hsa-miR-422a, hsa-miR-371a-5p, hsa-miR-203a-3p hsa-miR-16-5p, hsa-miR-15b-5p, hsa-miR-449b-5p, hsa-miR-431-5p hsa-miR-34c-5p	33	2

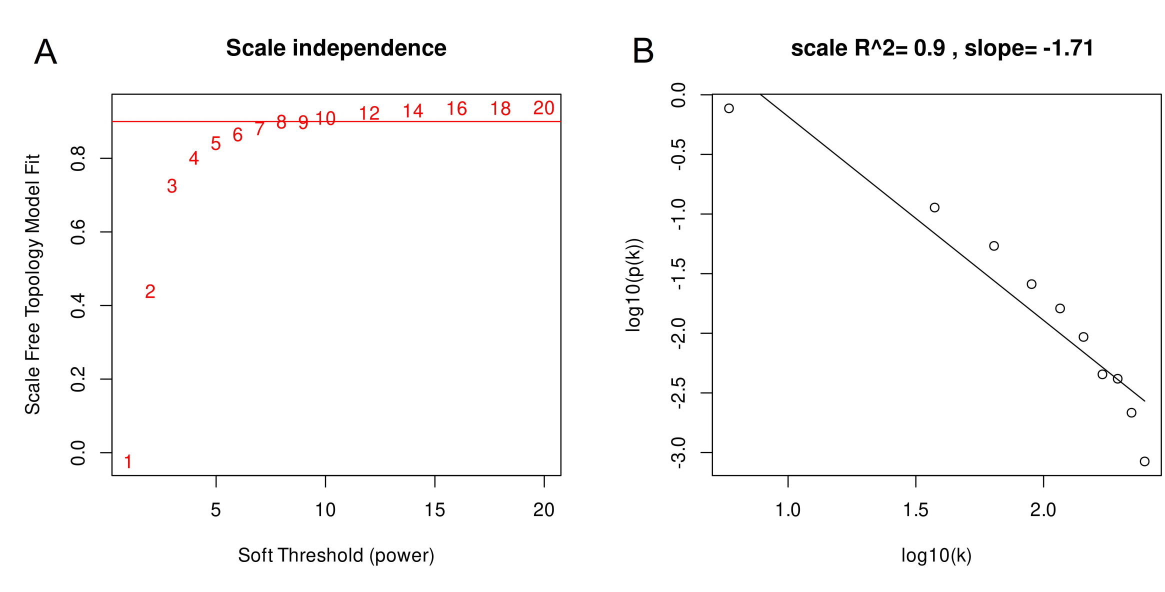

Supplementary Figure S1. Soft threshold selection to construct the gene co-expression network with topological analysis. A: Choose 10 as the optimized soft threshold to achieve favourable scale-free network topological distribution with the R^2 = 0.9. B: The co-expression network constructed with the optimized soft threshold of 10 satisfied favourable scale-free network topological distribution with the R^2 = 0.9.

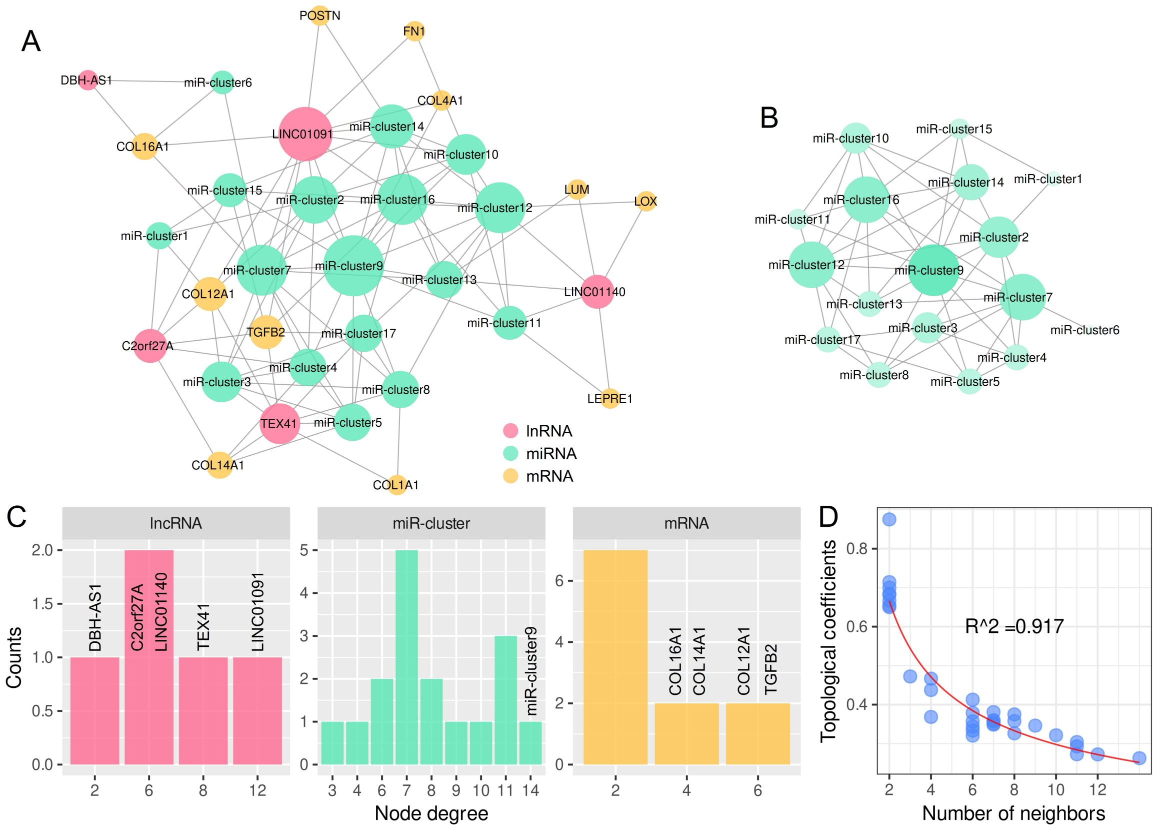

Supplementary Figure S2. LncRNA-miRcluster-mRNA network for 11 ARVC hub genes with topological analysis. A: The lncRNA-miRcluster-mRNA network, larger node sizes indicate higher degrees within the network. B: The overlaps among the miR-clusters. C: The distributions of the node degrees of lncRNA, miR-cluster, and mRNA respectively. D: The distribution of the topological coefficients indicating the favourable fitness of scale-free network feature.
